# Impact of delayed intervention following admission for small bowel obstruction: A contemporary analysis

**DOI:** 10.1016/j.sopen.2023.09.002

**Published:** 2023-09-12

**Authors:** Shannon Richardson, Nikhil L. Chervu, Russyan Mark Mabeza, Nam Yong Cho, Ayesha Ng, Arjun Verma, Amulya Vadlakonda, Syed Shahyan Bakhtiyar, Peyman Benharash

**Affiliations:** a Cardiovascular Outcomes Research Laboratories, Department of Surgery, David Geffen School of Medicine at UCLA, Los Angeles, CA, United States of America; b Department of Surgery, David Geffen School of Medicine at UCLA, Los Angeles, CA, United States of America; c Department of Surgery, University of Colorado Aurora, CO, United States of America

**Keywords:** Small bowel obstruction, Outcomes, Delay, Delayed intervention

## Abstract

**Background:**

The optimal timing of surgical intervention for small bowel obstruction (SBO) remains debated.

**Methods:**

All adults admitted for SBO were identified in the 2018–2019 National Inpatient Sample. Patients undergoing small bowel resection or lysis of adhesion after three days were considered part of the *Delayed* cohort. All others were classified as *Early*. Multivariable regressions were used to assess independent predictors of delayed surgical intervention as well as associations between delayed management and in-hospital mortality, major adverse events (MAE), perioperative complications, postoperative length of stay (LOS), hospitalization costs and non-home discharge.

**Results:**

Among 28,440 patients who met study criteria, 52.0 % underwent delayed intervention. Black race (AOR 1.19, 95 % CI 1.03–1.36, ref.: White) and Medicare coverage (AOR 1.16, 95 % CI 1.01–1.33, ref.: private payer) were associated with increased odds of delayed surgical management. While delayed intervention was not significantly associated with death (AOR 1.27, 95 % CI 0.97–1.68), it was linked to greater odds of MAE (AOR 1.30, 95 % CI 1.16–1.45) and several perioperative complications. The *Delayed* cohort also faced an incremental increase in postoperative LOS (+1.29 days, 95 % CI 0.89–1.70) and hospitalization costs (+$11,000, 95 % CI 10,000-12,000). Moreover, delayed intervention was linked to increased odds of non-home discharge (AOR 1.64, 95 % CI 1.47–1.84).

**Conclusions:**

Delay in surgical management following SBO is linked to inferior clinical outcomes and increased resource use. Our findings highlight the need to ensure proper timing of surgery for SBO as well as efforts to standardize these practices across all demographics of patients.

## Article summary

Several sociodemographic factors are independently linked with delayed surgical intervention (>3 days) following admission for small bowel obstruction (SBO), and such delay is associated with inferior clinical outcomes and greater resource use. The importance of these findings is they highlight the need to ensure proper timing of surgery for SBO as well as efforts to standardize practices across all demographics of patients.

## Introduction

Postoperative adhesions following abdominal and gynecological surgery are the leading cause of bowel obstruction in the United States [1]. Among such complications, small bowel obstruction (SBO) accounts for 16 % of annual surgical admissions and >$2 billion in healthcare expenditures [2]. Others have previously reported significant variation in the management approach to patients with adhesive SBO [[3], [4], [5]]. While most cases of SBO can be managed conservatively, there remains a paucity of evidence to guide the duration of such therapy before surgical intervention is needed. Moreover, delays in surgical intervention in patients who fail nonoperative management may be associated with worse outcomes [6].

Most recent guidelines from the World Society of Emergency Surgery recommend trials of nonoperative management not exceeding three days for uncomplicated SBO [3]. While successful nonoperative management may mitigate unnecessary surgery, failed attempts followed by delayed operation pose a high risk of morbidity and death in addition to increased healthcare resource utilization [3,7]. Furthermore, nearly a quarter of adhesive SBO cases treated with *nil* per os and non-invasive gastrointestinal decompression eventually require surgical treatment [8]. Thus, elucidating optimal timing for surgical intervention for SBO is particularly relevant.

In the present work, we used a nationally representative cohort of SBO patients to assess the association of operative timing with clinical outcomes and resource utilization. We hypothesized that delayed intervention following SBO would be associated with inferior clinical outcomes and increased resource utilization.

## Methods

### Data source and study cohort

This was a cross-sectional study using the 2018–2019 National Inpatient Sample (NIS). NIS is the largest publicly available, all-payer inpatient database in the United States that provides accurate estimates for about 97 % of all inpatient hospitalizations using a survey design. *International Classification of Diseases, Tenth Revision* (ICD-10) diagnosis and procedural codes were used to determine national and regional estimates of inpatient outcomes and costs. The study protocol was deemed exempt from full review by the Institutional Review Board at the University of California, Los Angeles.

**Fig. 1:**
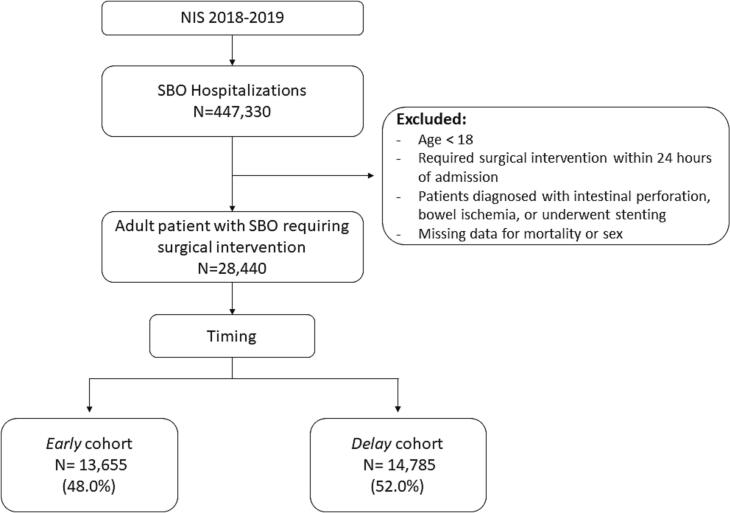
Consort diagram of patient selection criteria. NIS=National Inpatient Sample, SBO = small bowel obstruction.

Non-elective adult (≥18 years) hospitalizations with a primary diagnosis of SBO were identified using previously published ICD-10 codes [4]. Records with missing key information such as age, sex, and mortality were excluded (<0.1 %). Patients who were diagnosed with intestinal perforation or bowel ischemia, underwent stenting, or required surgical intervention within 24 h of admission were also excluded from further analysis (Fig. 1). Time to small bowel resection or lysis of adhesion was treated as a continuous variable.

### Variable and outcome definitions

**Fig. 2:**
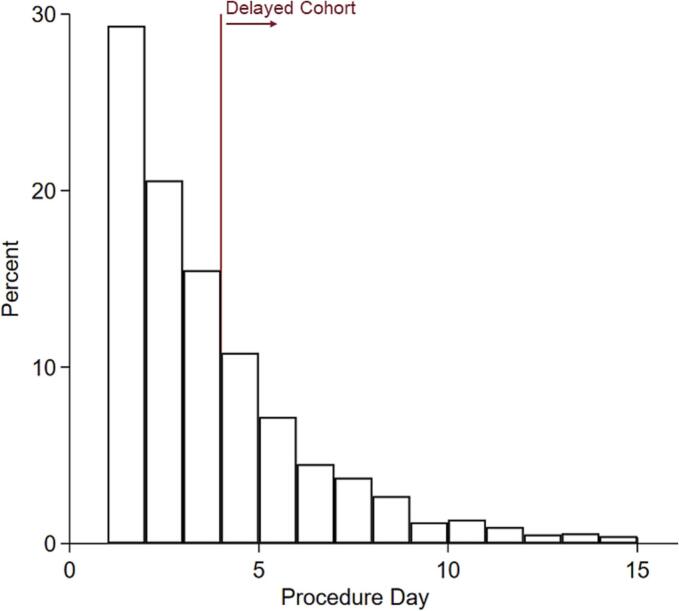
Distribution of procedure days following admission for small bowel obstruction (SBO) with the *Delayed* cohort defined as intervention at or after 4 days after hospitalization.

Patient and hospital level characteristics were defined according to NIS data dictionary. Variables of interest included age, sex, race, income quartile, insurance coverage (private, Medicare, Medicaid, self-pay/uninsured/other), and hospital teaching status. The van Walraven modification of the Elixhauser Comorbidity Index, a previously validated composite score of 30 comorbidities, was used to quantify patients' burden of chronic conditions [9]. Specific comorbidities were ascertained using ICD-10 codes. Major adverse events (MAE) were defined as a composite of in-hospital outcomes including mortality, mechanical ventilation >96 h, respiratory failure, sepsis, pneumonia, acute kidney injury, cardiovascular complications (cardiac arrest, cardiac tamponade, ventricular tachycardia or fibrillation) and thromboembolic events (pulmonary embolism and deep vein thrombosis). Hospitalization costs were calculated using hospital-specific cost-to-charge ratios provided by the Healthcare Cost and Utilization Project and adjusted for inflation to the 2019 Personal Health Index [10]. On exploratory analysis, median time to operation was identified as three days after hospitalization (Fig. 2). Patients undergoing small bowel resection or lysis of adhesion after three days were considered part of the *Delayed* cohort. All others were classified as *Early*.

The primary outcome was MAE while secondary endpoints included perioperative complications, hospitalization costs, postoperative length of stay (LOS), and non-home discharge.

### Statistical analysis

Categorical variables are reported as proportions (%) while continuous ones are summarized as means with standard deviations (SD) or medians with interquartile range (IQR) if non-parametric. The Pearson's chi-square and Adjusted Wald tests were used for bivariate comparisons between categorical and continuous variables, respectively.

Multivariate regressions with set NIS sampling weights were used to assess independent predictors of delayed surgical intervention. Regression models were also used to determine risk-adjusted associations between delayed surgery and outcomes of interest. The least absolute shrinkage and selection operator (LASSO) was used to select covariates included in regression models. Briefly, LASSO is a penalized regularization technique that eliminates collinear variables, reduces bias and enhances generalizability [11]. Models were evaluated using Akaike's and Bayesian Information Criteria in addition to receiver operating characteristics (C-statistic) as appropriate. Logistic and linear regressions are reported as adjusted odds ratios (AOR) and β coefficients with 95 % confidence intervals (95 % CI). All statistical analyses were conducted using Stata version 16.1 (StataCorp, College Station, TX) with significance set at an α of 0.05.

## Results

### Cohort and hospital characteristics

Among 28,440 patients with SBO requiring surgery, 14,785 (52.0 %) comprised the *Delayed* group. The median age was 69 years (IQR, 57–78) for both *Early* and *Delayed* cohorts (*P* = 0.4). Patients with delayed management were more commonly female (62.2 vs 59.0 %, *P* = 0.01), Black (17.6 vs 16.0 %), and had a higher burden of comorbidities (Table 1). Specifically, those in the delayed group were more often diagnosed with cardiac arrhythmias (24.0 vs 21.8 %, *P* = 0.05), cancer (11.7 vs 8.7 %, *P* < 0.001), and coagulopathy (5.6 vs 3.8 %, *P* = 0.001).

**Table 1:** Patient and hospital characteristics. Caption: All proportions reported as %. Continuous variables reported as median [interquartile range] unless specified otherwise.

	Early cohort (*n* = 13,655)	Delayed cohort (*n* = 14,785)	*P*-value
Age	69 [57–78]	69 [57–78]	0.4
Female	59.0	62.2	0.01
Elixhauser comorbidity index	3 [2–4]	3 [2–4]	<0.001
Race			0.03
White	69.9	69.6	
Black	16.0	17.6	
Hispanic	8.6	7.6	
Asian/PI	3.1	2.1	
Other	2.5	3.1	
Income quartile			0.3
76th–100	21.1	20.6	
51st–75th	25.7	24.9	
26th–50th	27.3	26.1	
0–25th	25.9	28.4	
Payer			0.15
Private	26.0	23.4	
Medicare	60.5	63.7	
Medicaid	8.2	7.9	
Uninsured/self	2.9	2.8	
Comorbidities			
Congestive heart failure	10.9	12.9	0.02
Coronary artery disease	12.9	13.0	0.9
Hypertension	56.7	54.9	0.2
Cardiac arrhythmias	21.8	24.0	0.05
Chronic pulmonary disease	17.8	18.2	0.7
Diabetes	17.3	17.7	0.7
Liver disease	3.4	4.6	0.02
Renal failure	10.8	12.4	0.06
PVD	8.4	9.1	0.3
Cancer	8.7	11.7	<0.001
Coagulopathy	3.8	5.6	0.001
Obesity	10.7	11.4	0.4
Smoker	14.0	11.7	0.007
Hospital region			0.001
Northeast	17.9	17.8	
Midwest	21.1	22.5	
South	39.5	42.5	
West	21.5	17.2	
Hospital size			<0.001
Small	24.8	20.5	
Medium	32.1	30.7	
Large	43.1	48.8	
Hospital teaching status	69.0	68.9	0.9
Hospital volume			0.7
Low	24.8	24.7	
Medium	45.5	46.6	
High	29.7	28.7	

### Predictors of delay

**Fig. 3:**
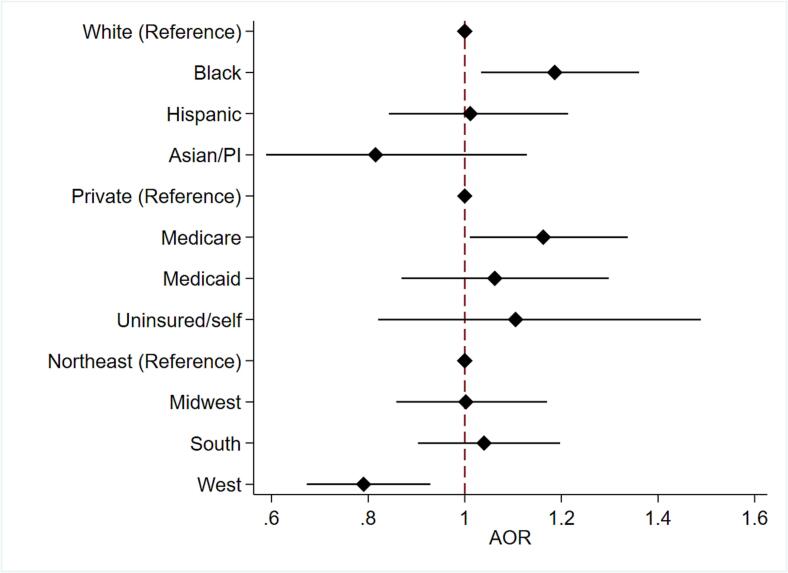
Risk-adjusted association between patient/hospital characteristics and delayed intervention following admission for small bowel obstruction. AOR = adjusted odds ratio.

After risk adjustment, Black race (AOR 1.19, 95 % CI 1.03–1.36, ref.: White) and Medicare coverage (AOR 1.16, 95 % CI 1.01–1.33, ref.: private payer) were associated with increased odds of delayed surgical management (Fig. 3). In contrast, patients with congestive heart failure (AOR 0.84, 95 % CI 0.70–0.99), hypertension (AOR 0.70, 95 % CI 0.63–0.79) and diabetes (AOR 0.81, 95 % CI 0.71–0.93) faced reduced odds of delayed intervention. Patients receiving treatment in the West had reduced odds of delayed intervention (AOR 0.79, 95 % CI 0.67–0.93, ref.: Northeast).

### Multivariable analyses

**Fig. 4:**
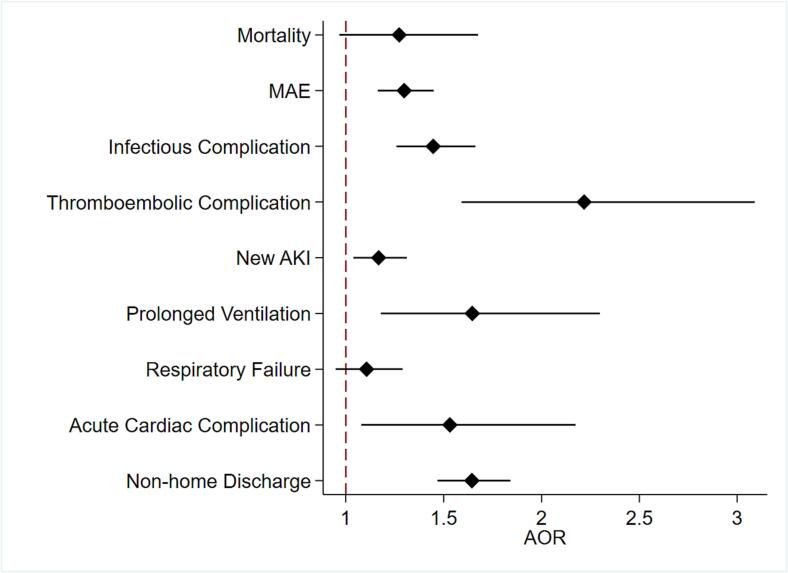
Risk-adjusted association between delayed intervention and clinical outcomes following admission for small bowel obstruction. AKI = acute kidney injury, AOR = adjusted odds ratio, MAE = major adverse event.

While delayed intervention was not significantly associated with mortality (AOR 1.27, 95 % CI 0.97–1.68), it was linked to greater odds of MAE (AOR 1.30, 95 % CI 1.16–1.45, Fig. 4). It was also linked to increased odds of prolonged mechanical ventilation (AOR 1.65, 95 % CI 1.18–2.30) as well as infectious (AOR 1.45, 95 % CI 1.26–1.66), thromboembolic (AOR 2.22, 95 % CI 1.59–3.09) and acute cardiac complications (AOR 1.53, 95 % CI 1.08–2.17, Fig. 4). The *Delayed* cohort also faced a 1.29-day increment in postoperative LOS (95 % CI 0.89–1.70) and incurred an additional $11,000 in costs (95 % CI 10,000-12,000). Moreover, delayed intervention was linked to increased odds of non-home discharge (AOR 1.64, 95 % CI 1.47–1.84).

## Discussion

Adhesive SBO is one of the most common surgical pathologies presenting emergently, requiring substantial healthcare resources and often resulting in significant morbidity and mortality [6]. While non-operative management is successful in 70–90 % of patients with adhesive SBO [12], appropriate timing of surgical intervention is generally poorly understood. In the present study, we noted a significant increase in major adverse events at 4 days following SBO admissions. Patients experiencing delay in either adhesiolysis or small bowel resection faced greater odds of perioperative complications, longer LOS, higher hospitalization costs, and increased non-home discharge. Furthermore, we noted several sociodemographic factors associated with delayed surgical intervention. Several of these findings merit further discussion.

Managing adhesive SBO is complex and requires a multidisciplinary approach, leaving appreciable room for variation in provider and institutional practice. Decisions regarding admitting service (medical vs surgical), duration of non-operative management trial, and surgical approach (open vs laparoscopic) all significantly influence clinical outcomes of SBO [4,5,7,[13], [14], [15]]. Surgery is indicated in the setting of bowel compromise and failed non-operative management [12], though the timing for operative intervention is less clearly defined in current literature. Several studies have concluded non-operative management to be safe for 72 hours [3,16], while others noted observation of adhesive SBO to be appropriate for no >5 days [4,17,18]. In the present study, we observed a 30 % increase in relative odds MAE in patients undergoing surgery 4 days after admission. This finding is consistent with a NSQIP study conducted by Keenan et al., which noted an increase in 30-day overall morbidity in patients experience a preoperative LOS >3 days [3]. Our findings suggest that surgical intervention within 3 days of admission is reasonable for most cases of uncomplicated SBO.

Patients who underwent delayed surgery in our study cohort faced a 30 % increase in relative odds of major adverse events. Texeira et al. reported similar findings, noting more than double the rate of mortality in the delayed operation group compared to their counterparts [7]. Furthermore, we observed higher adjusted rates of thromboembolic and infectious complications, among others, in the delayed cohort. Given the higher rates of adverse events, it is unsurprising that hospitalizations associated with delayed surgical intervention were significantly longer and more expensive. In the current value-based era of medicine, greater attention is being dedicated to optimizing outcomes while diminishing healthcare associated costs [19]. Of note, our analysis demonstrated an increment of $11,000 for patients who underwent delayed surgery. Such increase in healthcare expenditure underscores the significant financial implications of delayed surgical management. Altogether, our findings suggest that efforts to ensure appropriate surgical timing in SBO may benefit not only patients but hospital systems.

Given its associated inferior outcomes, factors influencing delay in surgery is particularly relevant. We noted several sociodemographic factors to be linked to delayed surgery, including Black race and public insurance coverage. Consistent with our findings, an analysis of nearly 14,000 SBO patients found that Black patients were significantly more likely to wait >5 days for surgery [17]. Another study found that Medicare- and Medicaid-covered patients faced greater odds of surgical delay [18]. The reason for such differences is complex and multifaceted, with structural, institutional and interpersonal factors likely at play. Differences in practice may also be influenced by geography. Of note, we found that patients in the West had a 20 % reduction in relative odds of delayed surgical intervention. More work is necessary to elucidate the impact of sociodemographic factors in decision making and operative planning for adhesive SBO.

This study has several important limitations due to its retrospective design and use of the NIS database. Due to its administrative nature, diagnoses and procedures are identified in NIS through ICD codes, which vary based on provider and hospital practice. Moreover, the study's retrospective design precludes any causal assumptions. Limited information regarding clinical factors, including electrolyte imbalances, abdominopelvic operations prior to the index admission and imaging studies are not available in NIS. Thus, potential unmeasured variables could have impacted both surgical delay and outcomes, leading to bias. Furthermore, we could not account for any decision to delay surgical intervention for SBO, which was likely influenced by hospital-level practices. Our analyses do not account for potential clustering within hospitals and future multi-level models would best explore this relationship. Due to a lack of granularity in ICD-10 coding for recurrent small bowel obstruction, a nuanced distinction that might exist between adhesive SBO and postoperative bowel obstructions could not be ascertained. Nonetheless, we utilized the largest available all-payer database and robust statistical methods to report on practice and real-world problems of surgical intervention related to SBO.

In conclusion, we found greater morbidity following surgical intervention for SBO at >3 days. Surgical delay is associated with inferior clinical outcomes and greater resource use. Several sociodemographic factors are independently linked to delayed surgical intervention. These findings highlight the necessity to ensure proper timing of surgery for SBO as well as efforts to standardize these practices across all demographics of patients.

## Study type

Retrospective cohort study.

## Ethical approval

Due to the deidentified nature of the NIS, this study was deemed exempt from full review by the Institutional Review Board at the University of California, Los Angeles. No attempts were made to deidentify patient information.

## CRediT authorship contribution statement

**Shannon Richardson**: Conceptualization, Methodology, Data Analysis, Writing Original Draft. **Nikhil Chervu**: Methodology, Writing, Reviewing, Submission Support **Russyan Mark Mabeza**: Methodology, Data Analysis, Validation, Writing, Reviewing, Generating Tables/Figures, Editing. **Nam Yong Cho, Ayesha Ng**: Writing, Reviewing, Editing, Generating Tables/Figures. **Arjun Verma**: Data analysis, Validation, Reviewing. **Amulya Vadlakonda, Syed Shahyan Bakhtiyar**: Methodology, Validation, Reviewing, Editing. **Peyman Benharash**: Conceptualization, Methodology, Supervision, Reviewing.

## Funding

The authors have no funding sources to report.

## Meeting presentation

Presented at the 18th Annual Academic Surgical Conference, Houston, February 2023.

## Declaration of competing interest

The authors of this manuscript have no related conflicts of interest to declare.
